# Acetaldehyde forms covalent GG intrastrand crosslinks in DNA

**DOI:** 10.1038/s41598-018-37239-6

**Published:** 2019-01-24

**Authors:** Yuina Sonohara, Junpei Yamamoto, Kosuke Tohashi, Reine Takatsuka, Tomonari Matsuda, Shigenori Iwai, Isao Kuraoka

**Affiliations:** 1 Graduate School of Engineering Science, Osaka University, 1-3 Machikaneyama, Toyonaka, Osaka, 560-8531 Japan; 2 Research Center for Environmental Quality Management, Kyoto University, 1-2 Yumihama, Ohtsu, Shiga, 520-0811 Japan; 3 Department of Chemistry, Faculty of Science, Fukuoka University, 8-19-1 Nanakuma, Jonan-ku, Fukuoka, 814-0180 Japan

## Abstract

Carcinogens often generate mutable DNA lesions that contribute to cancer and aging. However, the chemical structure of tumorigenic DNA lesions formed by acetaldehyde remains unknown, although it has long been considered an environmental mutagen in alcohol, tobacco, and food. Here, we identify an aldehyde-induced DNA lesion, forming an intrastrand crosslink between adjacent guanine bases, but not in single guanine bases or in other combinations of nucleotides. The GG intrastrand crosslink exists in equilibrium in the presence of aldehyde, and therefore it has not been detected or analyzed in the previous investigations. The newly identified GG intrastrand crosslinks might explain the toxicity and mutagenicity of acetaldehyde in DNA metabolism.

## Introduction

Acetaldehyde is a small, highly reactive compound that occurs naturally in various plants, ripe fruits, and vegetables, and is a key raw material in a wide range of chemical products. Importantly, humans appear to be constantly and unavoidably exposed to acetaldehyde, especially from alcohol, cigarettes, sugars, and polluted air^1^. Acetaldehyde is classified by the International Agency for Research on Cancer as having sufficient evidence of carcinogenicity in humans^1^. Indeed, carcinogenicity and mutagenicity have been repeatedly demonstrated in cells and experimental animals^2–4^.

Acetaldehyde is thought to cause a variety of DNA lesions in living cells^5,6^, but paradoxically, the major acetaldehyde-induced DNA lesions have very little effect on replication, because DNA polymerases bypass them in a nonmutagenic manner^7^. For example, acetaldehyde reacts with deoxyguanosine to form *N*^2^-ethylidenedeoxyguanosine, a type of Schiff base adduct^8,9^. This adduct is relatively unstable but abundant in the human liver, at about 0.1 per 10^6^ nucleotides, even in the absence of exposure to exogenous acetaldehyde^8,10^, suggesting that acetaldehyde is endogenously produced from normal metabolism. *N*^2^-ethylidenedeoxyguanosine can be stabilized by chemical reduction of the Schiff base to *N*^2^-ethyldeoxyguanosine^8^, which is then typically used as a model adduct in research. DNA repair pathways for these lesions have not been found^11^.

One hint for the identity of mutagenic lesions caused by acetaldehyde comes from a test of mutagenicity, in which a plasmid containing a selectable drug resistance gene is incubated with acetaldehyde *in vitro* and then transfected into human cells. This revealed an increase of GG to TT mutations in NER-deficient human XP cells^12^. However, the structure, physiological significance, and tumorigenicity of acetaldehyde-induced DNA lesions that elicit such mutations are unknown. We report here that acetaldehyde forms covalent guanine dimers. These lesions are specifically formed in adjacent deoxyguanosine residues, but not in single deoxyguanosine residues, or in deoxyadenosine, deoxycytosine, and thymidine residues. Detailed analysis revealed that acetaldehyde forms reversible intrastrand crosslinks in GG. These lesions are unstable, and hence have not been previously observed, but are likely to be toxic and mutagenic. We propose that these GG lesions may account for the apparent carcinogenicity of acetaldehyde in humans, and our understanding of these lesions may help develop new strategies to prevent alcohol-related cancer.

## Results

### Acetaldehyde reacts with oligonucleotides containing GG

**Figure 1:**
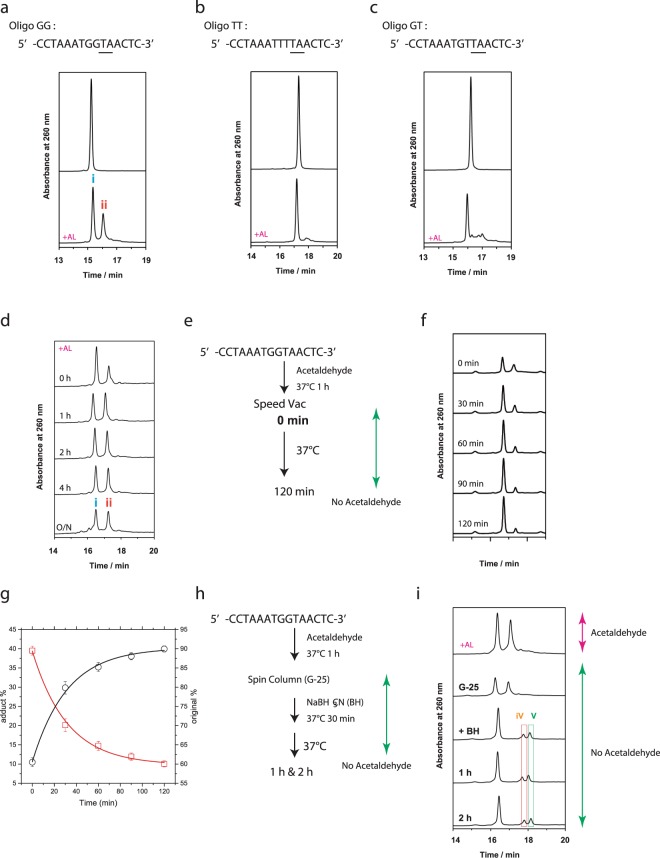
Acetaldehyde reacts with oligonucleotides containing GG, but not TT or GT. Oligonucleotides were incubated at 37 °C for 1 h in the presence of acetaldehyde (AL), and analyzed by HPLC to test the effects of acetaldehyde on (**a**) oligoGG (5′-CCTAAATGGTAACTC-3′), (**b**) oligoTT (5′-CCTAAATTTTAACTC-3′), and (**c**) oligoGT (5′-CCTAAATGTTAACTC-3′). (**d**) HPLC analysis of oligoGG reacted with acetaldehyde at 37 °C for 0 h, 1 h, 2 h, 4 h, and overnight. (**e**) Stability of acetaldehyde-treated oligonucleotides. OligoGG was incubated with acetaldehyde at 37 °C for 1 h, evaporated in a SpeedVac to remove acetaldehyde, incubated at 37 °C at pH 7, and (**f**) analyzed by HPLC. (**g**) Decrease of the acetaldehyde adduct (red) and increase of oligoGG (black) under physiological conditions. The decay and rise were globally fitted with a monoexponential function with a shared time constant. (**h**) Stabilization of acetaldehyde-treated oligonucleotides. OligoGG was incubated with acetaldehyde at 37 °C for 1 h, purified using a spin column to remove acetaldehyde, incubated with NaBH_3_CN at 37 °C for 30 min and for another 1 h or 2 h, and (**i**) analyzed by HPLC in the presence of NaBH_3_CN.

Acetaldehyde was previously reported to react with deoxyguanosine, deoxycytidine, and deoxyadenosine^13^, and to induce GG-to-TT tandem mutations in plasmid DNA^12^. Thus, a 15-mer synthetic oligomer with GG (oligoGG, Fig. 1a upper panel) was incubated with acetaldehyde at 37 °C for 1 h, and analyzed by HPLC. As shown in Fig. 1a, acetaldehyde generated a new peak (peak ii) in addition to the initial substrate (peak i), suggesting that DNA adducts were formed. As the oligomer contained not only GG, but also CC and AA sequences, oligomers with TT (oligoTT) or with only one G (oligoGT) were also tested. Strikingly, very little modified product was observed (Fig. 1b,c), indicating that acetaldehyde only reacts with GG sequences. Acetaldehyde also did not appear to react with oligonucleotides containing GA or AG (Supplementary Fig. 1a), GC or CG (Supplementary Fig. 1b), or GTG or GTTG (Supplementary Fig. 1c). Furthermore, we found that adducts were not formed under our experimental conditions even in GG dimers (Supplementary Fig. 2a,b), suggesting that acetaldehyde reacts only with GG sequences present in longer sequences. However, extended reaction times with oligoGG (Fig. 1d) also did not increase the amount of adducts formed after 1 h, suggesting that the reaction is reversible and reaches equilibrium.

We then investigated whether acetaldehyde reacts with complementary oligonucleotides that contain a GG site in one strand (Supplementary Fig. 3). As before, adducts were clearly formed in single-stranded oligonucleotides containing GG (oligoGG2, Supplementary Fig. 3a upper panel, peak iii), whereas broad peaks were observed in complementary strands (anti-oligoGG2, Supplementary Fig. 3b upper panel). Similarly, adducts were formed in oligoGG2 annealed to its complementary strand (ds-oligoGG2, Supplementary Fig. 3c upper panel), indicating that acetaldehyde reacts with both single-stranded and double-stranded DNA.

Acetaldehyde has been reported to produce interstrand crosslinks, especially in cells deficient in the Fanconi anemia pathway, which are notably hypersensitive to acetaldehyde^14,15^. However, we found that acetaldehyde-induced adducts were not formed under our experimental conditions in double-stranded oligonucleotides with a predicted GG interstrand crosslinking site (Supplementary Fig. 4), implying that acetaldehyde does not form interstrand crosslinks in this case.

In addition, we investigated whether a detectable human DNA glycosylase of BER might catalyze the cleavage in acetaldehyde-treated oligonucleotides containing GG using normal human cell extracts^16^. After incubation with HeLa cell extracts, no indications of any DNA glycosylase active on acetaldehyde-treated oligonucleotides were obtained under our experimental conditions, whereas DNA strand cleavage of a control oligonucleotide containing a uracil residue was detected (Supplementary Fig. 5).

### Acetaldehyde-induced DNA lesions are reversible

We attempted to purify the product formed by acetaldehyde and oligoGG to determine its structure. Because the boiling point of acetaldehyde is 20.2 °C, it was easily removed from reaction mixtures by SpeedVac. However, removal of acetaldehyde unexpectedly reduced the amount of adducts (peak ii) and increased the amount of the initial substrate (peak i, Supplementary Fig. 6), even when stored at −20 °C for 24 h (Supplementary Fig. 6). This observation indicated that adducts reverted to the unreacted state in the absence of acetaldehyde. Indeed, the adducts completely disappeared after incubation at 75 °C or 95 °C for 5 min (Supplementary Fig. 7). The reversion of the adducts was analyzed in detail under physiological conditions after the removal of acetaldehyde by SpeedVac (Fig. 1e). Upon incubation of the mixture at 37 °C at pH 7.0, decrease in the adducts and increase in the original oligonucleotide were observed (Fig. 1f) with a time constant of 31.6 ± 2.3 min (Fig. 1g), and the reaction reached at equilibrium after 120 min. These results highlight the instability of adducts formed by acetaldehyde, and suggest that the reaction between oligonucleotides and acetaldehyde is reversible.

We hypothesized that the adduct is unstable because of imine structures formed between acetaldehyde and NH_2_ in deoxyguanosine. Hence, oligos that reacted with acetaldehyde were purified by gel filtration and immediately incubated for 30 min with NaBH_3_CN, a strong reducing agent, to isolate a stable structure. Samples were then observed for another 1–2 h to monitor stability (Fig. 1h). Notably, incubation with NaBH_3_CN for 30 min–2 h resolved the adduct peak (peak ii) into peaks iv and v (Fig. 1i).

### Mass analysis of reduced acetaldehyde-reacted oligonucleotides

**Figure 2:**
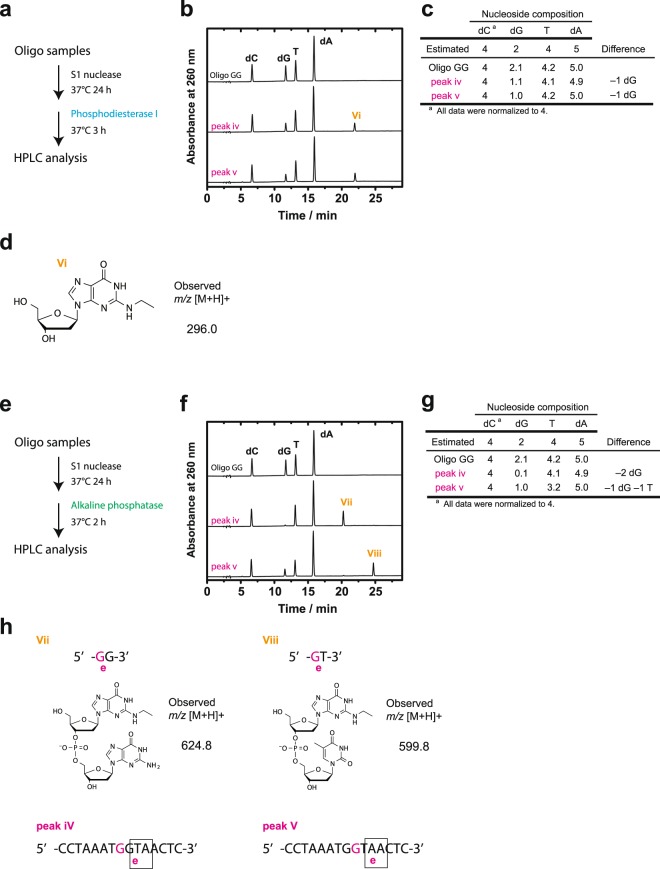
Mass spectrometry of acetaldehyde-treated oligonucleotides containing a GG site. (**a**) Experimental scheme. Aldehyde-treated oligonucleotides (peaks iv and v) were purified in the presence of NaBH_3_CN, digested with S1 nuclease at 37 °C for 24 h and then with phosphodiesterase I at 37 °C for 4 h, and (**b**) analyzed by HPLC. (**c**) Nucleoside composition of peaks iv and v. (**d**) Estimated structure and observed m/z [M + H]^+^ in peak vi. (**e**) Acetaldehyde-treated oligonucleotides (peaks iv and v) were also purified in the presence of NaBH_3_CN, digested with S1 nuclease at 37 °C for 24 h and then with alkaline phosphatase at 37 °C for 2 h, and (**f**) analyzed by HPLC. (**g**) Nucleoside composition of peaks iv and v. (**h**) Estimated structure and observed m/z [M + H]^+^ in peaks vii and viii. Predicted oligonucleotides are indicated in peaks iv and v.

We then analyzed the nucleosides produced by digesting peaks iv and v with a nuclease and a phosphatase. Remarkably, the same peak (peak vi) was detected when peaks iv and v (Fig. 2b) were digested with S1 nuclease and phosphodiesterase I (Fig. 2a). Compositional analysis indicated that one deoxyguanosine was lost (Fig. 2c and Supplementary Fig. 8). Furthermore, a compound with m/z [M + H]^+^ 296.0 was detected by LC-MS, indicating that the product is *N*^2^-ethyldeoxyguanosine, which has theoretical m/z 295.29, as shown in Fig. 2d. These results suggest that peaks iv and v are somehow chemically different, even though they each contain *N*^2^-ethyldeoxyguanosine. Hence, we partially digested both peaks using S1 nuclease and an alkaline phosphatase (Fig. 2e), obtaining peaks vii and viii from peaks iv and v, respectively (Fig. 2f). Compositional analysis indicated that two deoxyguanosines were lost in peak iv, and one deoxyguanosine and one thymine were lost in peak v (Fig. 2g and Supplementary Fig. 9). Mass analysis also detected a compound with m/z [M + H]^+^ 624.8 in peak vii and 599.8 in peak viii (Fig. 2h), indicating that the products were *N*^2^-ethyldeoxyguanosine attached to deoxyguanosine in peak vii and *N*^2^-ethyldeoxyguanosine attached to thymine in peak viii. The structures of oligonucleotides in peaks iv and v, which were generated by reducing acetaldehyde-reacted oligoGG, are illustrated in Fig. 2h, lower panel.

### Chemical structure of acetaldehyde-induced intrastrand GG crosslinks

**Figure 3:**
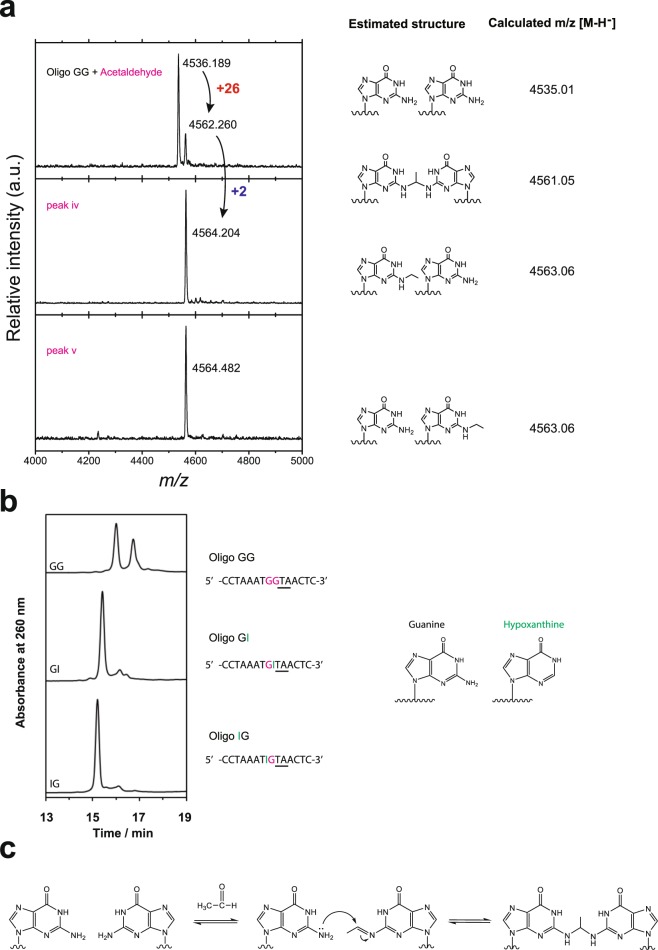
Acetaldehyde induces intrastrand crosslinking of GG sequences. (**a**) Estimated structure and observed m/z [M + H]^+^ of oligonucleotides reacted with acetaldehyde (AL). (**b**) Chemical structure of guanine and NH_2_ -free hypoxanthine. HPLC analysis of oligoGG, GI, and IG reacted with acetaldehyde. (**c**) A model of the chemical reaction between two guanines and acetaldehyde.

As noted, reduction of acetaldehyde-reacted oligoGG with NaBH_3_CN produced oligonucleotides containing one *N*^2^-ethyldeoxyguanosine at the 5′ or 3′ end of a GG sequence. However, products with two *N*^2^-ethyldeoxyguanosines at the GG site were not observed, suggesting that acetaldehyde may have formed intrastrand crosslinks that were subsequently reduced by NaBH_3_CN. To test this possibility, we analyzed acetaldehyde reaction mixtures by MALDI-TOF-MS. As shown in Fig. 3a, a product with m/z [M + H]^+^ 4562.26 was observed, indicating that acetaldehyde formed intrastrand-crosslinked GG, which has theoretical m/z 4561.05. In addition, the observed m/z [M + H]^+^ of products in peaks vi and v correspond with the calculated m/z [M + H]^+^ of expected oligonucleotides as shown in Fig. 3a, demonstrating conclusively that acetaldehyde forms intrastrand-crosslinked GG.

The NH_2_ group in deoxyguanosine seems to be important in forming such a structure. Therefore, we tested whether acetaldehyde generates intrastrand crosslinks in hypoxanthine, a guanine without NH_2_ (Fig. 3b). Reactions with oligoGG, oligoGI, and oligoIG (Fig. 3c) indicated that NH_2_ in deoxyguanosine is essential for crosslinking.

## Discussion

In this study, we found that acetaldehyde specifically reacts with single-stranded or double-stranded oligonucleotides containing GG, but not TT, GT, GA, AG, GC, or CG. However, the lesions formed are unstable, and produce two stable products containing *N*^2^-ethyldeoxyguanosine only when reduced with NaBH_3_CN. Finally, mass analysis revealed that acetaldehyde forms GG intrastrand crosslinks, a new type of DNA lesion that is uniquely reversible.

### Chemical features

The putative mechanism of intrastrand-crosslinking is illustrated in Fig. 3c. In this reaction, an imine is formed between acetaldehyde and the amino group of one of the guanine bases, which then undergoes nucleophilic attack by the amino group of the adjacent guanine base. Imine formation is known to be reversible in aqueous solutions, and Fig. 1d,f, and Supplementary Fig. 6 demonstrate that crosslinking after imine formation is also in equilibrium and splits the product into imine and amine intermediates. This mechanism is supported by mass spectrometry (Figs 2 and 3). Each of the two products obtained in the presence of NaBH_4_ contained an ethyl group, which was presumably formed by the reduction of the imine intermediate, whereas the crosslinked product was successfully detected only in the absence of reducing agent. Interestingly, intrastrand crosslinking was not observed by Wang *et al*.^8^, who investigated interstrand crosslinking by acetaldehyde, likely because the reversibly intrastrand-crosslinked products were too labile to be detected. In addition, we believe that intrastrand crosslinking is more likely to occur than interstrand crosslinking, which requires two acetaldehyde molecules, and is rate-limited by the addition of the second acetaldehyde, as also observed during aldol condensation in alkaline conditions.

Many examples of imine formation between aldehydes and amino groups in guanine have been reported^8,17^. We note that the amino group in 2′-deoxyguanosine has a lower pK_a_ value (2.5) than 2′-deoxyadenosine and 2′-deoxycytidine (3.8 and 4.3, respectively)^18^. As the nucleophilicity of a more basic compound is lower than that of a less basic group in an aqueous solution due to hydration, imine formation is more favorable at a guanine base than at others. Furthermore, we assume that consecutive reactions at GG sites stabilize the resulting adduct, as intrastrand crosslinks were observed only at such sites (Fig. 1 and Supplementary Fig. 1), and not in isolated G sites (Fig. 1c and Supplementary Fig. 1a,b).

### DNA repair and mutagenesis

Acetaldehyde-induced GG intrastrand crosslinks are most likely repaired by the versatile NER pathway. Indeed, an intrastrand-crosslinked GG lesion probably resembles a UV-induced TT dimer and thus distorts the DNA helix. Consequently, such a distortion can only be resolved by NER, which primarily repairs bulky helix-distorting damage from environmental mutagens, and not by BER, which repairs non-bulky and non-distorting DNA modifications from endogenous and some chemical carcinogens. Indeed, cleavage by DNA glycosylase during BER would not remove this lesion, because guanine would remain attached to another guanine even after cleavage of the glycosyl bond. In contrast, Matsuda *et al*. previously reported that acetaldehyde-treated DNA increases GG-to-TT mutations in NER-deficient cells, which are also more sensitive to acetaldehyde than NER-proficient cells^12^. And an acetaldehyde-induced GG intrastrand crosslink also resemble a cis-diammineplatinum(II)-induced GG intrastrand crosslink that are repaired by NER, in chemical GG interstrand crosslink products^19^. In addition, since many distorted DNA lesions (e.g. a UV-induced TT dimer and a cis-diammineplatinum(II)-induced GG intrastrand crosslink) have been shown to block transcription, an acetaldehyde-induced GG intrastrand crosslink might induce a transcription arrest by RNA polymerase II which is trigger of transcription-coupled NER^20^. However, to demonstrate this directly, oligonucleotides containing acetaldehyde-induced crosslinks would have to be purified and assayed by *in vitro* NER using human cell extracts. Although such experiments are challenging to execute because of the chemical instability of these lesions, there might be the possibility to observe the biological effects of the lesions using a structurally stable analog of intrastrand crosslink lesion.

Stepwise reaction of two molecules of acetaldehyde with DNA *in vivo*^8^ produces two other possible DNA adducts: *R*- and *S*-α-CH_3_-γ-OH-1,*N*^2^-propano-2′-deoxyguanosine^21^. These adducts are thought to be repaired by NER, and to induce G-to-T transversion 5–10% of the time, although Hoogsteen base pairing with cytosine is also possible^22,23^.

In addition, these DNA lesions may react with deoxyguanosine on the opposite strand to form an interstrand crosslink, or with protein to form a DNA-protein crosslink. To resolve the spectrum of structures formed, repair pathways for such lesions are probably complex and versatile.

### XP and Fanconi anemia

NER is impaired in XP^24^; as a result, cells from patients with XP are hypersensitive to UV light. NER-deficient XPA cells were also previously reported to be more sensitive to acetaldehyde than normal cells, although XPA knockout chicken DT40 cells were only slightly more sensitive than control cells^15^. These results imply that NER resolves acetaldehyde-induced GG intrastrand crosslinks. In any case, even low concentrations of acetaldehyde, such as those typically found in the human body, may induce genomic DNA lesions because the reaction is reversible. Thus, similar to cyclopurine lesions formed during oxidative stress, this lesion may be an endogenous DNA lesion that accelerates neurodegeneration in XP.

Fanconi anemia is an inherited genomic instability disorder caused by mutations in genes regulating replication-dependent removal of interstrand DNA crosslinks^25^. Accordingly, in response to genotoxicity, the Fanconi anemia DNA repair pathway is thought to coordinate a complex mechanism combining elements of homologous recombination, NER, and translesion DNA synthesis. Notably, cells from patients with Fanconi anemia are hypersensitive to exogenous interstrand crosslinking agents such as mitomycin C and cisplatin, as well as to endogenous acetaldehyde^6,15^, which we found to form intrastrand crosslinks rather than interstrand crosslinks. Nevertheless, all types of lesions may block DNA synthesis during replication, thereby causing spontaneous cell death. Indeed, cells from patients with Fanconi anemia are sensitive to not only crosslinking agents but also noncrosslinking agents^26^, UV^27^, camptothecin^28^, and etoposide^29^ in some cases.

Intriguingly, recent genome-wide analysis of sequence signatures indicates that CC-to-AA mutations are associated with cancer, as are GG lesions^30^. This survey also suggests that mutations are induced on transcribed strands, and are linked to transcription-coupled NER. Thus, we propose that the GG-to-TT mutations observed in cancers may in part be correlated with acetaldehyde-induced intrastrand crosslinks.

## Methods

### HPLC

Samples were analyzed by HPLC on a gradient-type analytical HPLC system (Gilson, Inc.) equipped with a Waters 2996 photodiode-array detector. Oligonucleotides were loaded on a µBondasphere C18 column (Waters Co.) at 1.0 mL min^−1^ and 30 °C, and eluted over a linear, 20-minute gradient of 6–14% acetonitrile in 0.1 M triethylammonium acetate pH 7.0. In contrast, nucleosides from digested oligonucleotides were loaded on an Inertsil ODS-3 column (GL Science Inc.) at 1.0 mL min^−1^ and ambient temperature, and eluted over 30 min along a linear gradient of 2.5–20% acetonitrile in 0.1 M triethylammonium acetate, pH 7.0.

### Stability of acetaldehyde-crosslinked products

Purified oligonucleotides were allowed to react with acetaldehyde at 37 °C for 1 h in water, and the solution was freeze-dried by SpeedVac for 1 h. The dried material was dissolved in 100 mM phosphate buffer (pH 7.0) and incubated at 37 °C. Aliquots (5 µL) of the mixture was sampled at appropriate intervals and immediately analyzed by HPLC, with a linear 15-min gradient of 7–14% acetonitrile in 0.1 M triethylammonium acetate pH 7.0. The yields of the product were estimated using the peak areas of the original material and crosslinked product detected at 260 nm. The experiments were independently performed in triplicate, and the results were globally fitted with a monoexponential function with Origin2016.

### MALDI-TOF mass spectrometry

Acetaldehyde (10 µL) was reacted at room temperature for 1 h with 10 µM 15-mer oligo with GG, which had been prepared in 100 µL of water. An aliquot (2 µL) of the reaction mixture was spotted on a predried 3-hydroxypicolinic acid matrix, dried a second time in ambient conditions, and analyzed on a Bruker Ultraflex III MALDI TOF/TOF mass spectrometer. MALDI TOF mass spectra of purified unreacted oligonucleotides were also collected with the same instrument and matrix.

### Enzymatic digestion of oligonucleotides

Purified oligonucletides (1 nmol) were mixed with 180 U of S1 nuclease (TaKaRa Bio) in 20 µL of 30 mM sodium acetate buffer pH 4.6 containing 100 mM NaCl and 1 mM ZnCl_2_. After 24 h at 37 °C, the product was digested for another 4 h at 37 °C in 30 µL of 0.167 M Tris-HCl buffer pH 7.0 containing 5 U of antarctic phosphatase or 40 U of phosphodiesterase I from *Crotalus adamanteus* venom. Finally, digests were stored at –80 °C until HPLC analysis.

For partial digestion, purified oligonucleotides were first treated with S1 nuclease as previously described. After 24 hours, the reaction was quenched with 5 µL of 0.5 M Tris-HCl buffer pH 9.0 containing 10 mM MgCl_2_, and mixed with 2 µL of alkaline phosphatase from *Escherichia coli* C75 (1 U, TaKaRa Bio) and 23 µL of water. The mixture (50 µL) was incubated at 37 °C for 2 hours, and stored at −80 °C until HPLC analysis.

## Supplementary information

Supplementary Info
