# Supplementary material for: Acetaldehyde forms covalent GG intrastrand crosslinks in DNA
Source: Sci Rep. 2019 Jan 24;9:660. doi: 10.1038/s41598-018-37239-6 (PMC6345987; doi:10.1038/s41598-018-37239-6)
Supplement: Supplementary file 1 — Supplementary Info [file 41598_2018_37239_MOESM1_ESM.docx]

**Supplementary Information**

**Acetaldehyde forms covalent GG intrastrand crosslinks in DNA**

Yuina Sonohara^1^, Junpei Yamamoto^1^, Kosuke Tohashi^1^, Reine Takatsuka^1^, Tomonari Matsuda^2^, Shigenori Iwai^1^, and Isao Kuraoka^1^*

^1^Graduate School of Engineering Science, Osaka University, 1-3 Machikaneyama, Toyonaka, Osaka 560-8531, Japan. ^2^Research Center for Environmental Quality Management, Kyoto University, 1-2 Yumihama, Ohtsu, Shiga 520-0811, Japan.

***Corresponding author:** Isao Kuraoka, Graduate School of Engineering Science, Osaka University, 1-3 Machikaneyama, Toyonaka, Osaka 560-8531, Japan. Tel: +81-6-6850-6240, Fax: +81-6-6850-6240, E-mail: kuraoka@chem.es.osaka-u.ac.jp


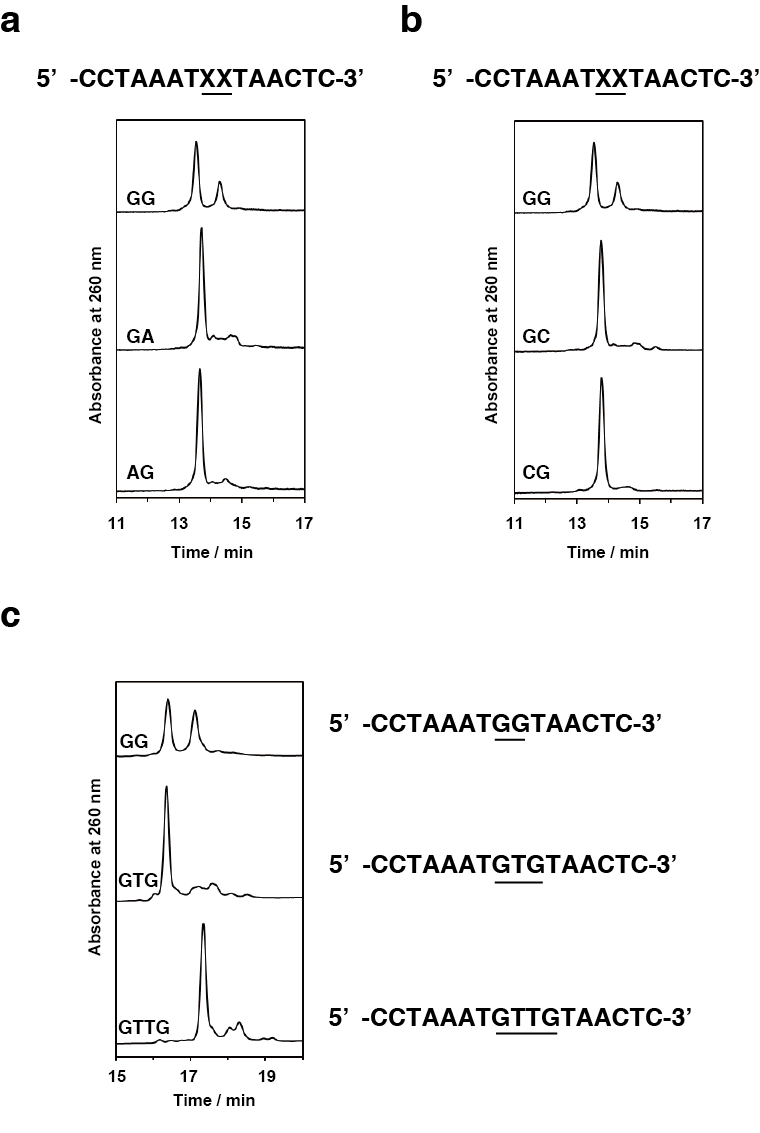


**Figure S1** Acetaldehyde reacts with oligonucleotides containing GG, but not GA, AG, GC, GC, GTG and GTTG. Oligonucleotides were incubated at 37°C for 1 h in the presence of acetaldehyde. HPLC analysis of the oligonucleotides (a) Oligo:5’-CCTAAATXXTAACTC-3’, XX=GG, GA, AG, (b) Oligo:5’-CCTAAATXXTAACTC-3’, XX=GG, GC, CG, and (c) Oligo (each right panel, GG, GTG, GTTG in the presence of acetaldehyde.


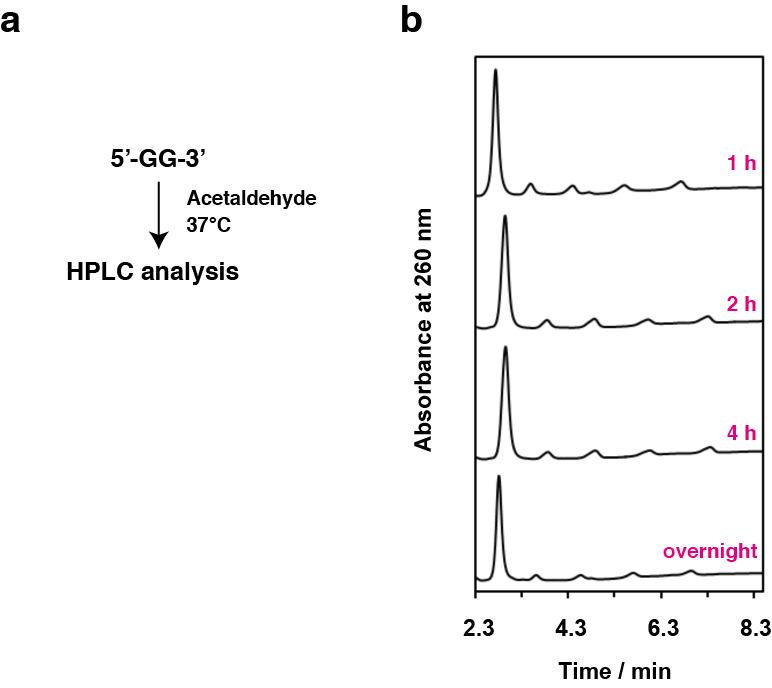


**Figure S2** Acetaldehyde reacts with di-nucleotide GG (2-mer) oligonucleotides. (a) Scheme for HPLC analysis of the acetaldehyde-treated di-nucleotides. Oligonucleotides were incubated at 37°C in the presence of acetaldehyde. (b) HPLC analysis of the acetaldehyde-treated di-nucleotide in the time-dependent manner. di-nucleotides in the presence of acetaldehyde were incubated for the indicated time (1 h, 2 h, 4 h, and overnight).


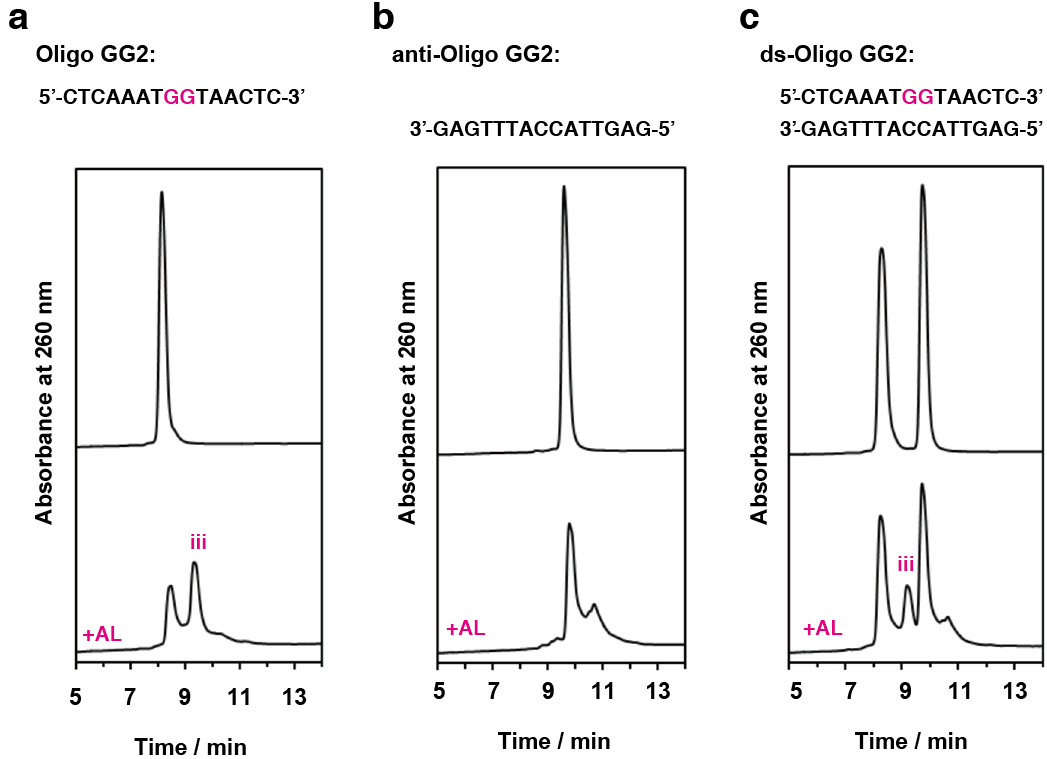


**Figure S3** Acetaldehyde reacts with single strand (ss) or double strand (ds) oligonucleotides containing a GG sequence. The oligonucleotides were incubated at 37°C for 1 h in the presence of acetaldehyde. (a) HPLC analysis of the ss-oligonucleotides (Oligo GG2: 5’-CTCAAATGGTAACTC-3’) in the presence of acetaldehyde. (b) HPLC analysis of the ss-oligonucleotides (anti-Oligo GG2: 3’-GAGTTTACCATTGAG-5’) in the presence of acetaldehyde. (c) HPLC analysis of the ds-oligonucleotides containing a GG sequence in the presence of acetaldehyde.


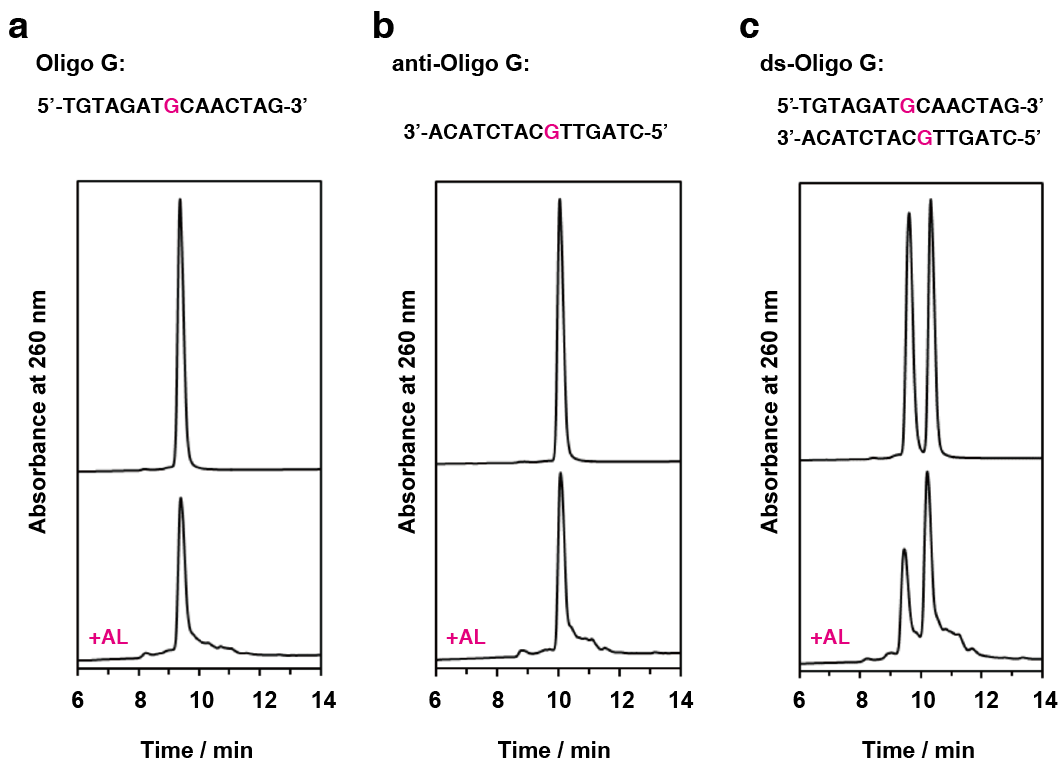


**Figure S4** Acetaldehyde reacts with single strand (ss) or double strand (ds) oligonucleotides containing a predicted GG interstrand crosslinks sequence. The oligonucleotides were incubated at 37°C for 1 h in the presence of acetaldehyde. (a) HPLC analysis of the ss-oligonucleotides (Oligo G: 5’-TGTAGATGCAACTAG-3’) in the presence of acetaldehyde. (b) HPLC analysis of the ss-oligonucleotides (anti-Oligo GG2: 3’-ACATCTACGTTGATC-5’) in the presence of acetaldehyde. (c) HPLC analysis of the ds-oligonucleotides containing a predicted GG interstrand crosslinks sequence in the presence of acetaldehyde.

**
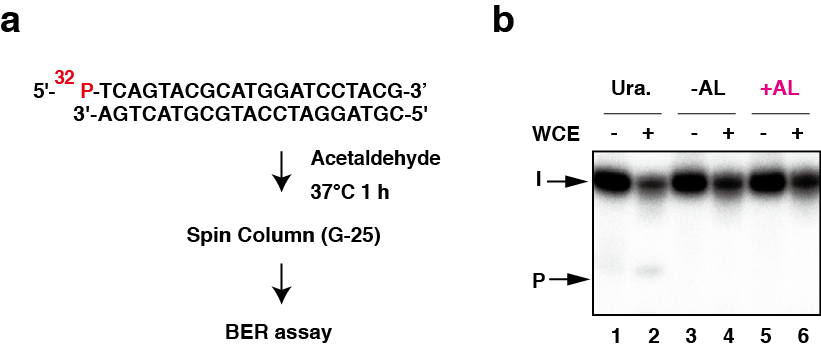
**

**Figure S5** DNA glycosylase assay of acetaldehyde-treated oligonucleotides containing GG sequence. (a) Scheme for DNA glycosylase assay of the acetaldehyde-treated oligonucleotides. Oligonucleotides were incubated at 37°C for 1 h in the presence of acetaldehyde and purified using a G-25 spin column. The oligonucleotides were incubated with HeLa cell extracts (WCE). (b) DNA glycosylase assay. Lanes 1 and 2, duplex oligonucleotide containing a uracil instead of GG sequence at the same position; lanes 3 and 4, duplex oligonucleotide without acetaldehyde-treatments; lanes 5 and 6, duplex oligonucleotide with acetaldehyde-treatments. These oligonucleotides were incubated with (lanes 2, 4, and 6) or without (lanes 1, 3, and 5) HeLa cell extract (WCE) at 37°C for 30 mim and then at 95°C for 5 min. The samples were treated with AP endonuclease I (APE1) at 37°C for 5 mim. The arrows mark the positions of intact oligonucleotide (I), and product (P) after DNA glycosylase action followed by APE1 treatment.


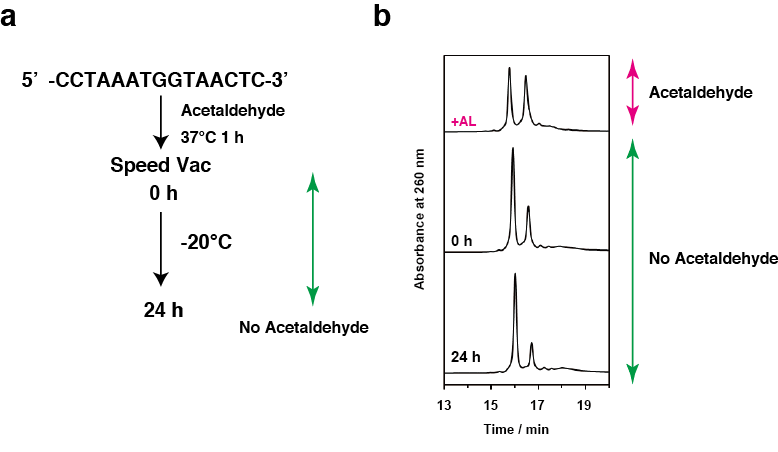


**Figure S6** Stability of acetaldehyde-treated oligonucleotides. (**a**) OligoGG was incubated with acetaldehyde at 37 °C for 1 h, evaporated in a SpeedVac to remove acetaldehyde, stored at -20 °C, and (**b**) analyzed by HPLC.


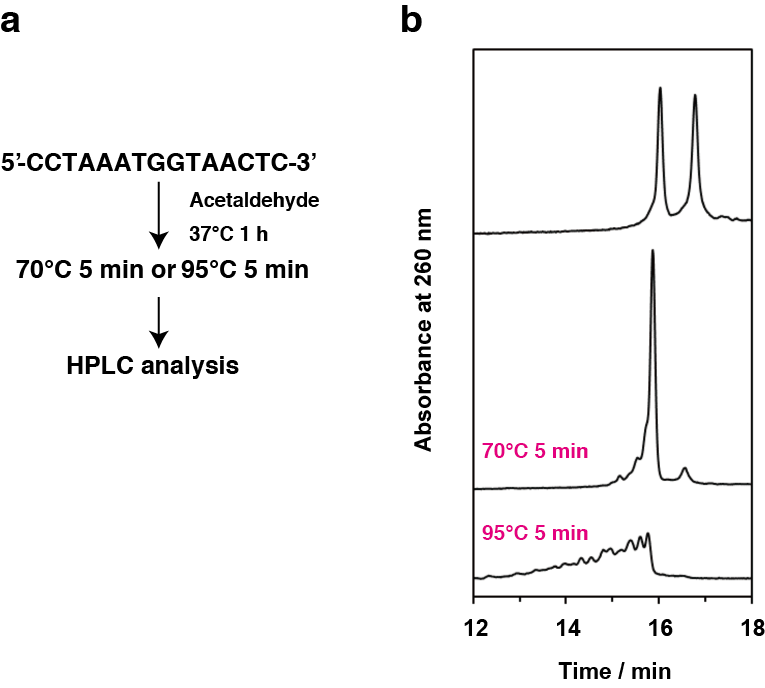


**Figure S7** Thermal stability of acetaldehyde-treated oligonucleotides containing GG sequence. (a) Scheme for HPLC analysis of the acetaldehyde-treated oligonucleotides. Oligonucleotides were incubated at 37°C for 1 h in the presence of acetaldehyde and additionally incubated at 70°C for 5min or 95°C for 5 min. (b) HPLC analysis of the heat-treated each oligonucleotide.


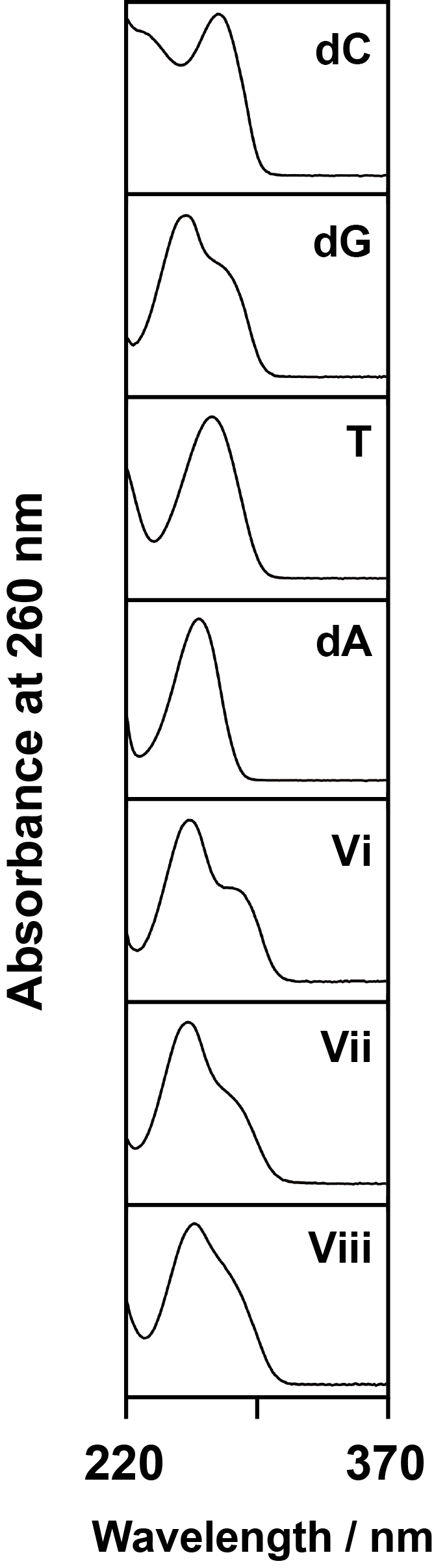


**Figure S8** Absorbance of digested nucleosides from oligonucleotides products in peak iv and peak v. New peaks (vi, vii, viii) from digested nucleosides were analyzed.
